# Dominant resistance against plant viruses

**DOI:** 10.3389/fpls.2014.00307

**Published:** 2014-06-27

**Authors:** Dryas de Ronde, Patrick Butterbach, Richard Kormelink

**Affiliations:** Laboratory of Virology, Department of Plant Sciences, Wageningen UniversityWageningen, Netherlands

**Keywords:** *R* genes, PTI, PAMP-triggered immunity, ETI, RNAi, avirulence, hypersensitive response

## Abstract

To establish a successful infection plant viruses have to overcome a defense system composed of several layers. This review will overview the various strategies plants employ to combat viral infections with main emphasis on the current status of single dominant resistance (*R*) genes identified against plant viruses and the corresponding avirulence (*Avr*) genes identified so far. The most common models to explain the mode of action of dominant *R* genes will be presented. Finally, in brief the hypersensitive response (HR) and extreme resistance (ER), and the functional and structural similarity of *R* genes to sensors of innate immunity in mammalian cell systems will be described.

## Introduction

During the past decades it has become clear that plants have a unique and complex defense system that consists of several layers, which enables them to avoid, suppress, or actively defend against pathogens from all kingdoms like fungi, bacteria, nematodes, and viruses. Of all plant viruses known, only a few cause serious diseases and, if so, mostly limited to a very small number of crops. In general, most viruses have a limited (natural) host range and the number of so-called non-hosts exceeds those of hosts. In those plants that are hosts, viruses encounter different mechanisms of defense. Some act general against all viruses and this response is part of the innate immune system, while others are virus-specific and involve resistance genes. Triggering of the latter simultaneously mediates a rapid necrosis at the site of virus entry and prevents further spread of the virus throughout the host. In several cases resistance genes do not confer absolute resistance and low levels of virus replication can still be observed. In those cases the genes are referred to as partial resistance genes or tolerance genes.

While throughout the years reviews on resistance genes have appeared with regular intervals, these mostly had their main focus on fungal and bacterial resistance genes, primarily due to the large amount of data available. This review aims to present an overview on the current status of resistance genes against plant viruses, with emphasis on single dominant resistance genes. The very basis of plant pathogens (among others plant viruses) not being able to infect all plants is due to a mechanism called non-host resistance (NHR) (For an extensive review on this, see Uma et al., 2011). NHR holds for all plant pathogens and is a generic, nonspecific resistance that can be divided into two main types, distinguished by the mechanism and mode of recognition (Mysore and Ryu, 2004). Type 1 is the most pre-dominant type of NHR and presents a basic defense mechanism that prevents pathogen invasion, e.g., thickening of the cell-wall, secondary metabolite production, etc. This type of resistance usually is symptomless. In contrast, type 2 NHR is associated with induction of necrosis at the site of infection, and is induced when pathogens overcome type 1 resistance. Here, the pathogen is recognized through specific structures or proteins that are associated with the pathogen. The recognition of these structures/proteins, so called microbe associated molecular patterns (MAMPs) or PAMPS (Pathogen), takes place by pattern recognition receptors (PRRs) on plant plasma membranes. These PRRs recognize conserved structures of pathogens, like flaggelin from the flagella of bacteria or chitin from the cell wall of fungi, and induce a so called PAMP triggered immunity (PTI) response (Jones and Dangl, 2006). Since plant viruses need to overcome the physical barrier of a cell wall, they enter their host cells either via mechanical inoculation or the infection is mediated by vectors like insects, nematodes, or even fungi. Direct recognition of viruses probably does not occur in the apoplast. However, a study recently reported on the possible involvement of (intracellular) receptor-like kinases (RLKs), of the like that are involved in PAMP recognition by PRRs, in plant-virus interactions (Kørner et al., 2013).

One of the first innate immune responses all plant viruses encounter when invading a host consists of antiviral RNA silencing [also called RNA interference (RNAi) and in the very early days post-transcriptional gene silencing (PTSG)]. RNA silencing is a host response triggered by double stranded (ds)RNA. These molecules thus act as a MAMP/PAMP and in which RNAi can be regarded as PTI. The main difference with pathogens such as fungi and bacteria is that recognition of viral MAMPs/PAMPs occurs intracellularly (Ding and Voinnet, 2007).

RNA silencing consists of two major “branches”; the first one is that of small-interfering (si)RNAs, and one of the hallmarks for antiviral RNAi, and the second one is that of (host-gene encoded) micro (mi)RNAs involved in gene regulation. The antiviral RNAi response is induced by viral double stranded (ds)RNA molecules that arise from replicative intermediates or secondary RNA folding structures. These structures are sensed by a host RNase type III-like enzyme called Dicer-like (DCL) protein and cleaved into short interfering (si)RNA of 21–24 nucleotides (nt) in size (Sharma et al., 2013). The siRNAs generated are unwound and only one strand, the so-called guide-strand, is uploaded into a functional protein complex termed RNA-induced silencing complex (RISC). This activated complex next surveils and subsequently degrades (viral) RNA target molecules with sequence complementarity to the guide-strand. Degradation of the target RNA is mediated by slicer, the core component of RISC, which is represented by a member of the Argonaut (AGO) family of proteins (Vaucheret, 2008; Sharma et al., 2013). After primary siRNAs have been generated, in plants an amplification of siRNAs follows, which is required to mount an RNAi response to effectively combat virus infections locally and systemically. This amplification involves host RNA dependent RNA polymerases (RDRs) that are able to convert (aberrant) viral (m)RNAs into dsRNA in a siRNA-dependent and -independent manner (Csorba et al., 2009). Their subsequent processing by DCL leads to the generation of secondary siRNAs that correspond to sequences outside the primary target sequence, a process also called transitive silencing (Sijen et al., 2001). The antiviral RNAi response acts against all RNA and DNA viruses (Incarbone and Dunoyer, 2013), but in general is a relatively slow process that does not lead to complete clearance of viral infections. For an extensive description of RNAi readers are referred to nice reviews from e.g., Ding (2010) and Sharma et al. (2013).

Besides RNAi, viruses may also run into another, second layer of defense that involves resistance genes. While most of these are triggered by and confer resistance to a specific virus only, some act against several (related) viruses. The major class of these genes represent single dominant resistance genes (and of which the biggest group consists of the NB-LRR type), while others are recessive, tolerance, or partial resistance genes. A very nice example of a dominant resistance gene of the latter case has recently been described with the cloning and characterization of the *Ty-1* resistance gene from tomato against *Tomato yellow leaf curl* geminivirus (TYLCV) This gene encodes an RNA-dependent RNA polymerase (RdRp) and is proposed to confer resistance to TYLCV by amplifying the RNAi signal (Verlaan et al., 2013). Tomato plants containing *Ty-1* do not show symptoms upon a challenge with TYLCV, but low levels of virus can still be detected.

Recessive resistance (Truniger and Aranda, 2009) acting against viruses, relies on the observation that viruses require host factors (also called susceptibility factors) to enable an infection. The inability of interaction between such host factor and the virus leads to resistance. Since susceptibility factors are dominant, a resistance based on these requires all gene copies to be in the (resistant) recessive state. This explains why such resistance is generally termed recessive resistance. The majority of the recessive resistance genes known against plant viruses have been reported for potyviruses (Kang et al., 2005) and encode translation initiation factors of the 4E or 4G family (eIF4E/eIF4G) (Truniger and Aranda, 2009). The latter proteins need to interact with the cap-structure on (viral) transcripts, to allow for translation. Potyviral transcripts do not contain a cap structure, but provide a VPg (Virus-protein genome linked) to render their transcripts translatable in a cap-independent manner. Potyvirus infection leads to host shut off of cap-dependent transcripts, but only allow the cap-independent transcripts to be translated mediated by a subgroup of translation initiation factors; eIF(iso)4E/G. Viruses that encode their own cap-like structure (like potyviruses: VPg) require interaction with the translation initiation factors eIF4E/eIF4G for translation, this in turn induces a selection pressure on the host to escape the interaction between VPg and eIF4e, leading to recessive resistance. Recessive resistance genes toward other pathogens, such as fungi and bacteria have only been described to a limited extent and their encoded susceptibility factors (*S* genes) are proposed to provide a more durable resistance than dominant *R* genes. However due to their functions they may cause pleiotropic effects when knocked out from the host genome (Gawehns et al., 2013).

## Dominant resistance

### Effector-mediated triggering of single dominant resistance genes

**Figure 1:**
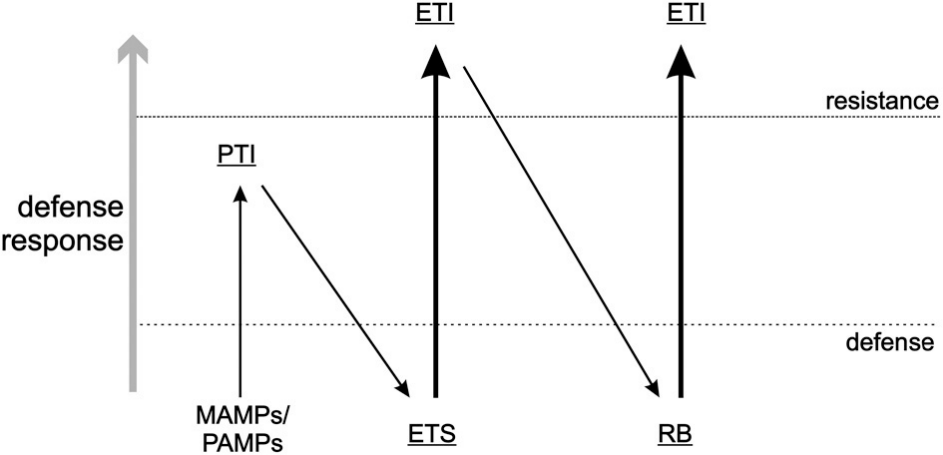
**Zig-zag-model**. A visual presentation of the arms race between pathogen and host according to Jones and Dangl (2006). Here, a slightly modified version of that model is presented and as described in this review. MAMPs/PAMPs, Microbe/Pathogen associated molecular patterns; PTI, PAMP triggered immunity; ETS, Effector triggered susceptibility; ETI, Effector-triggered immunity; RB, resistance breaker.

Plant pathogens need to evade or suppress the PTI response in plants and achieve the latter by encoding effector proteins that can interfere with the recognition by PRRs, usually by binding to the substrate that PRRs would otherwise recognize. This process allows the pathogen to establish a successful infection, and is referred to as Effector Triggered Susceptibility (ETS) (Figure 1): a strategy that also applies to antiviral RNAi. One of the most common strategies plant viruses use to counteract RNAi is to encode RNA silencing suppressors (RSS), viral proteins that interfere with a specific part of the RNAi pathway and thereby reduce its effectiveness against plant viruses (Burgyan and Havelda, 2011). The majority of plant virus RSS proteins exert this activity through binding of small interfering (si)RNAs, or sometimes (also) long dsRNA, and thereby prevent their uploading into RISC and Dicer-cleavage, respectively (Lakatos et al., 2006). In recent years some RSS have also been discovered to inhibit the RNAi pathway in other ways, e.g., by binding directly to key-enzyme proteins like AGO1, the core component of RISC during the antiviral RNAi response (Zhang et al., 2006; Giner et al., 2010). Viral suppression of RNAi leads to a stage of ETS during which viruses are able to establish a successful infection.

Single dominant resistance (*R*) gene products (in)directly sense the presence of a specific pathogen by their effector, termed avirulence factors (Avr), as a counter defense against ETS, leading to a stage called Effector-Triggered Immunity (ETI) (Figure 1). Triggering of *R* genes is generally associated with a (concomitant) induction of a programmed cell death response, as visualized by the rapid appearance of necrotic lesions (a hypersensitive response, HR) or in rare occasions extreme resistance (ER) during which no necrosis is observed at all. However, more and more evidence is presented, that there is an uncoupling of the resistance response from the programmed cell death response, although both can work in concert. Due to these responses, viruses (and other pathogens) are confined to the site of entry/invasion where infections are prevented. In contrast to the slower onset of antiviral RNAi, the *R* gene response generally is rapid and within ~3/4 days lead to containment of the virus.

Dominant *R* genes basically can be grouped into two classes, namely those that encode NB-LRRs and all others. The major class of *R* genes consists of the NB-LRR type and encode proteins that, irrespective of the pathogen they recognize, consist of three domains; (1) the Nucleotide Binding Site (NBS) in the center of the protein, (2) a Leucine Rich Repeat (LRR) at the C-terminal end, and (3) a Coiled-coil (CC) or Toll and Interleukin-1 Receptor (TIR) domain at the N-terminal end of the resistance gene product (Moffett, 2009). The LRR determines the specificity of the target protein and is the most variable part of the protein, therefore considered to be under selection pressure to evolve for recognition of (new) target proteins. The NBS is composed of a conserved part that contains the Nucleotide Binding site (NB) and an ARC-domain, both required to bind and hydrolyze ATP. *R* genes that contain an N-terminal TIR domain are only found in dicots from the angiosperms (Collier et al., 2011), and through this domain share homology to Toll-like receptor (TLR) proteins, that act as PRRs in the innate immunity response in animal systems. Those with no predicted structure at its N-terminus, are grouped with the CC-domain (Maekawa et al., 2011a; Hao et al., 2013) in the non-TIR group. All three domains are involved in an interaction with each other and change conformationally upon activation to subsequently induce the resistance response (Lukasik and Takken, 2009; Slootweg et al., 2010).

Only a few cases have been described in which the dominant *R* gene product recognizes an Avr protein through direct interaction (Jia et al., 2000; Deslandes et al., 2003; Dodds et al., 2006; Krasileva et al., 2010; Chen et al., 2012; Cesari et al., 2013), of which one is the TMV-p50 helicase domain (Ueda et al., 2006). In the majority of known *R* genes recognition of the pathogen occurs indirectly and involves host proteins, which are considered guardees, decoys, or baits, depending on the model, as further discussed below (Model of *R* Gene Recognition) (Van Der Biezen and Jones, 1998; Jones and Dangl, 2006; Van Der Hoorn and Kamoun, 2008; Collier and Moffett, 2009).

### Cloned *R* genes and their known Avr determinants

While for fungi and bacteria many resistance genes have been cloned and characterized, resistance genes against plant viruses have received growing interest during the last two decades, still only few of the latter have been cloned so far. Table S1.1 gives an up-to-date summary of all *R* genes against plant viruses, known or currently under investigation. For some of these genes the viral *Avr* determinant has been identified. From this large, extensive list of *R* genes (>200), only 22 have been cloned and characterized. Some *R* genes have functional alleles in other plant species, often showing a similar Avr recognition. The majority of the known *R* gene products are of the CC-NB-LRR type, whereas only a small group belongs to the TIR-NB-LRR group (Table 1).

**Table 1:** **Cloned dominant resistance genes against plant viruses, organized into the NB-LRRs and the non-NB-LRRs, and their Avr determinants (when identified)**.

**Plant host**	***R* gene**	**Type: NB-LRR**	**Recognizes**	**Virus genus**	**AVR**	**References**
*Arabidopsis thaliana*	*HRT*	CC-NB-LRR [HR]	TCV [*Turnip crinkle virus*]	*Carmovirus*	CP	1, 2
Mouse ear cress						
	*RCY1*	CC-NB-LRR [HR]	CMV [*Cucumber mosaic virus*]	*Cucumovirus*	CP	3–6
*Brassica campestris*	*BcTuR3*	TIR-NBS-LRR	TuMV [*Turnip mosaic virus*]	*Potyvirus*	Unknown	17, 18
Field mustard						
*Capsicum annuum frutescens chinense chacoense*	*L-locus: L^*1*^ L^2^ L^3^ L^4^*	CC-NB-LRR	TMV [*Tobacco mosaic virus*] by *L^1234^*	*Tobamovirus*	CP (all)	25, 31–34, 43–45
Pepper			ToMV [*Tomato mosaic virus*] by *L^1234^*			
			TMGMV [*Tobacco mild green mosaic virus*] by *L^1234^*			
			BPeMV [*Bell pepper mottle virus*] by *L^1234^*			
			PaMMV [*Paprika mild mottle virus*] by *L^234^*			
			ObPV [*Obuda pepper virus*] by *L^234^*			
			PMMoV [*Pepper mild mottle virus*] by *L^34^(isolate dependent)*			
*Glycine max*	*Rsv1* (locus)	CC-NB-LRR [ER/HR]	SMV [*Soybean mosaic virus*]	*Potyvirus*	P3+ HC-Pro	65–69
Soybean						
*Cucumis melo*	*Pvr1*	TIR-NB-LRR	PRSV [*Papaya ringspot virus*]	*Potyvirus*	Unknown	50, 288
*Muskmelon*	*Pvr2*				Unknown	
*Nicotiana glutinosa*	*N*	TIR-NB-LRR [cell-cell mov.]	TMV [*Tobacco mosaic virus*]	*Tobamovirus*	p50 [Helicase]	105–111
Tobacco						
*Phaseolus vulgaris*	*I* (locus)	TIR-NB-LRR [ER/HR/phloem necr.]	BCMV [*Bean common mosaic virus*]	*Potyvirus*	Unknown	127–133
Kidney bean			BNMV [*Bean necrotic mosaic virus*]			
			BICMV *[Blackeye cowpea mosaic virus]*			
			AzMV *[Azuki mosaic virus]*			
			CABMV [*Cowpea aphid-borne mosaic virus*]			
			PWV [*Passionfruit woodiness virus*]			
			SMV [*Soybean mosaic virus*]			
			ThPV [*Thailand passiflora virus*]			
			WMV [*Watermelon mosaic virus*]			
			ZYMV [*Zucchini yellow mosaic virus*]			
	*PvVTT1*	TIR-NB-LRR [HR]	BDMV [*Bean dwarf mosaic virus*]	*Begomovirus*	BV1 (NSP)	134–139
	*PvCMR1* (*RT4-4*)	TIR-NB-LRR [syst. necrosis]	CMV [*Cucumber mosaic virus*]	*Cucumovirus*	2a	156
*Poncirus trifoliate*	*Ctv* (locus)	CC-NB-LRR	CTV [*Citrus tristeza virus*]	*Closterovirus*	Unknown	158–160
Trifoliate orange						
*Solanum peruvianum*	*Sw5b*	CC-NB-LRR [HR]	TSWV [*Tomato spotted wilt virus*] and other tospoviruses	*Tospovirus*	NSm	179–183
Tomato						
	*Tm-2*	CC-NB-LRR [HR]	TMV [*Tobacco mosaic virus*]	*Tobamovirus*	30 kD MP	171, 188, 189
			ToMV [*Tomato mosaic virus*] and other tobamoviruses			
	*Tm-2^2^*	CC-NB-LRR [HR]	ToMV [*Tomato mosaic virus*]	*Tobamovirus*	30 kD MP	171, 190–193
			TMV [*Tobacco mosaic virus*] and other tobamoviruses			
*Solanum tuberosum*	*Rx1*	CC-NB-LRR [ER/HR]	PVX [*Potato virus X*] and other potex viruses	*Potexvirus*	CP	195, 198, 199, 230–234
Potato						
	*Rx2*	CC-NB-LRR	PVX [*Potato virus X*]	*Potexvirus*	CP	138, 232
	*Y-1*	TIR-NB-LRR	PVY [*Potato virus Y*]	*Potyvirus*	Unknown	237, 238
*Vigna mungo*	*CYR1*	CC-NB-LRR	MYMV [*Mungbean yellow mosaic virus*]	*Begomovirus*	CP	256, 257
Black gram						
**Plant host**	***R* gene**	**Type: non NB-LRR**	**Recognizes**	**Virus genus**	**AVR**	**References**
*Arabidopsis thaliana*	*JAX1*	Jacalin-like [lectin gene]	*Broad resistance against potexvirus*	*Potexvirus*	Unknown	258
Mouse ear cress						
	*RTM1*	Jacalin-like [prev. syst. mov.] [RTM3 not cloned]	TEV [*Tobacco etch virus*]	*Potyvirus*	CP	7–9
	*RTM2*		PPV [*Plum pox virus*]		CP	
	RTM3		LMV [*Lettuce mosaic virus*]		CP	
*Solanum chilense*	*Ty-1*	RDR [Tol.]	TYLCV [*Tomato yellow leaf curl virus*]	*Begomovirus*	No	30, 166, 167
Tomato	*Ty-3*					
*Solanum hirsutum*	*Tm-1*	TIM-barrel-like domain protein [ER] [Replication]	ToMV [*Tomato mosaic virus*]	*Tobamovirus*	Replicase Helicase-domain	169–174
Tomato						

1, Cooley et al., 2000; 2, Ren et al., 2000; 3, Takahashi et al., 2001; 4, Takahashi et al., 2002; 5, Takahashi et al., 2004; 6, Sekine et al., 2006; 7, Chisholm et al., 2000; 8, Whitham et al., 2000; 9, Decroocq et al., 2009; 17, Provvidenti and Hampton, 1992; 18, Ma et al., 2010; 25, Moury and Verdin, 2012; 30, Grube et al., 2000; 31, Tomita et al., 2008; 32, Tomita et al., 2011; 33, Matsumoto et al., 2008; 34, Sawada et al., 2004; 43, De La Cruz et al., 1997; 44, Holmes, 1937; 45, Berzal-Herranz et al., 1995; 50, Anagnostou et al., 2000; 65, Hayes et al., 2004; 66, Hajimorad and Hill, 2001; 67, Hajimorad et al., 2005a; 68, Wen et al., 2013; 69, Eggenberger et al., 2008; 105, Whitham et al., 1994; 106, Erickson et al., 1999; 107, Baker et al., 1995; 108, Dinesh-Kumar et al., 1995; 109, Dinesh-Kumar et al., 2000; 110, Padgett and Beachy, 1993; 111, Padgett et al., 1997; 127, Vallejos et al., 2006; 128, Ariyarathne et al., 1999; 129, Collmer et al., 2000; 130, Kelly et al., 1995; 131, Kyle et al., 1986; 132, Fisher and Kyle, 1994; 133, Fisher and Kyle, 1996; 134, Zhou et al., 2007; 135, Garrido-Ramirez et al., 2000; 136, Seo et al., 2004; 137, Seo et al., 2007; 138, Wang et al., 1999; 139, Gururani et al., 2012; 156, Seo et al., 2006; 158, Yang et al., 2003; 159, Rai, 2006; 160, Harper et al., 2010; 166, Hanson et al., 2000; 167, Verlaan et al., 2013; 169, Ishibashi et al., 2007; 170, Ishibashi et al., 2012; 171, Pelham, 1966; 172, Yamafuji et al., 1991; 173, Meshi et al., 1988; 174, Kato et al., 2013; 179, Finlay, 1953; 180, Holmes, 1948; 181, Brommonschenkel et al., 2000; 182, Hallwass et al., 2014; 183, Hoffmann et al., 2001; 188, Hall, 1980; 189, Meshi et al., 1989; 190, Weber et al., 1993; 191, Lanfermeijer et al., 2003; 192, Lanfermeijer et al., 2005; 193, Tanksley et al., 1998; 195, Cockerham, 1970; 198, Solomon-Blackburn and Barker, 2001b; 199, Solomon-Blackburn and Barker, 2001a; 230, Bendahmane et al., 1995; 231, Bendahmane et al., 1999; 232, Bendahmane et al., 2000; 233, Baures et al., 2008; 234, Querci et al., 1995; 237, Vidal et al., 2002; 238, Zvereva and Pooggin, 2012; 256, Maiti et al., 2012; 257, Pal et al., 1991; 258, Bijaisoradat and Kuhn, 1985; 287, Yamaji et al., 2012; 288, Brotman et al., 2013.

A few dominant *R* genes against viruses have been described that do not belong to the NB-LRR type of genes, e.g., *RTM1*, *RTM2*, and *RTM3*. Latter resistance genes have been identified from *A. thaliana* and prevent the systemic spread of several potyviruses. In those cases the virus is not able to upload into the phloem to systemically disseminate into the host. In addition, there is also no induction of HR or production of salicylic acid (SA), as commonly observed with NB-LRR mediated resistance responses (Cosson et al., 2012). No direct interaction occurs between the RTM proteins with the potyvirus CP (Avr) protein. A resistance gene recently identified is *JAX*, a lectin gene that resembles the RTM gene based resistance and works broadly against potexviruses in *A. thaliana*, indicating an important role for lectins in plant immunity (Yamaji et al., 2012). Another type of a distinct *R* gene is *Tm-1*, found in the wild tomato species *S. hirsutum*, encoding a protein that contains a TIM-barrel. This barrel binds the replication proteins of *Tomato mosaic virus* (ToMV) and thereby inhibits RNA replication (Ishibashi et al., 2007). Also here, no typical NB-LRR type-associated response, like HR, is induced. Many homologs of *Tm-1* are found in other organisms from different kingdoms, like fungi, archae, and bacteria, suggesting that this gene (originally) presents a housekeeping gene (Ishibashi et al., 2012). Both *RTM* and *Tm-1* seem to play a role in the inhibition of a specific step required for successful infection by the virus. Whether these present a new class of dominant resistance genes remains to be determined.

From only a 1/3 of the total number of *R* genes directed against plant viruses, the virus Avr determinant is identified (Tables 1 and S1.1). Interestingly, functionally quite different viral proteins act as Avr determinants. Several *R* genes belong to the same locus (for instance the L-proteins in *Capsicum* spec.) or clearly act as homologs (Rx1 and Rx2 from *S. tuberosum*) and recognize the same Avr protein from overlapping virus species, indicating that these conserved R proteins are able to recognize similar structures but with an adapted spectrum (Bendahmane et al., 1995, 2000; Moury and Verdin, 2012). For several viruses, their corresponding *R* genes have not been identified yet, but their single dominant nature is deduced from the observation that an HR is being triggered. In some of these cases, the viral gene responsible for the induction of resistance, as indirectly monitored by HR, has been identified.

As described before and clear from Table 1, many different viral proteins can act as Avr determinants; whether it is the coat protein (e.g., *L*-locus from *Capsicum* against Tobamoviruses), the movement protein (e.g., *Tm-2*/*Tm-2*^2^ from tomato against Tobamoviruses), the replicase protein (e.g., *Tm-1* from tomato against *Tobacco mosaic virus*) or the RNAi suppressor protein (e.g., *HRT* from *A. thaliana* against *Turnip crinkle virus*), all potentially can act as elicitor of resistance (Meshi et al., 1989; Ishibashi et al., 2012; Moury and Verdin, 2012). Interestingly, for a majority of cases the ability to induce the resistance, as monitored by visual HR, could be uncoupled from the endogenous function of the viral protein but exceptions exist.

While the function of a viral protein is not a selective criterium to act as Avr-determinant, the “Zig-zag-model” by Jones and Dangl (2006) (Figure 1) implies that ETI (R gene mediated resistance) is a response to ETS and governed by effectors, i.e., molecules that act as virulence factors and contribute to (enhance) pathogen fitness. It is obvious that in case of RNAi as a PTI response against viruses, viral Avr proteins containing RSS activity contribute to virus fitness as a result of PTI suppression and thereby initiating ETS. On the other hand, some viral Avr proteins lack RSS activity which would indicate that effectors not necessarily would have to suppress PTI (RNAi) to contribute to virulence, as observed with several bacterial effectors. Unless, instead of RNAi, another innate immune response is being counteracted that is triggered via the activation of different intracellular PAMP receptors (e.g., in analogy to animal TLRs, see below).

### Model of *R* gene recognition

Although the mode of action of resistance genes still remains a matter of debate, models have been proposed for the triggering of the largest and most studied group of the NB-LRR type of dominant *R* genes. One of the most commonly accepted models is the “guard hypothesis” (Van Der Biezen and Jones, 1998; Jones and Dangl, 2006). In this model the resistance gene product guards a certain host protein, the “guardee,” and perceives alterations of this protein upon interaction with the Avr determinant to subsequently initiate a resistance response. It is possible that multiple *R* genes guard the same guardee, possibly *vice versa* as well, which thereby broadens the resistance spectrum of (a limited number of) *R* genes to a wide range of various pathogens; e.g., Rx1 and GPa2 both interact with the same guardee RanGAP2 (Tameling and Baulcombe, 2007; Moffett, 2009). Unfortunately, this model does not explain how resistance breaking virus isolates maintain their virulence. For this reason, alternative models have been postulated. According to the “decoy model” (Van Der Hoorn and Kamoun, 2008), a (proteinaceous) decoy evolved to act as a molecular sensor to only detect a pathogen without having any other role in the household machinery of the host. The “bait and switch model” and the similar “mousetrap model” have been more recently postulated and proposes that the *R* gene product in an “OFF” state forms a complex together with the guardee/decoy protein, that upon interaction of the Avr protein with the complex leads to a conformational switch (“ON”) and activates a downstream signaling pathway leading to resistance (Collier and Moffett, 2009; Lukasik and Takken, 2009). Recent studies on the resistance gene *Rx* from potato against PVX have shown that indeed intramolecular interactions keep the R gene product in an inactive state, while interaction of the effector protein releases these interactions and thereby activating the resistance downstream (Bendahmane et al., 2002; Moffett et al., 2002; Lukasik and Takken, 2009; Slootweg et al., 2013).

The downstream mechanism after resistance induction still remains unclear. However, one described case of the *R* gene from tobacco, the *N* gene, has revealed some of the downstream ways of controlling virus replication and obtaining resistance. The *N* gene encodes a TIR-NB-LRR protein and confers resistance against TMV and, upon transient co-expression with the p50 elicitor (helicase), an HR is induced in *N. tabacum*, a response that does not occur in *N. benthamiana*. Bhattacharjee et al. (2009) employed this observation in a series of experiments to dissect and assign downstream signaling of defense responses, related to the *R* gene. The studies indicated that the *N* gene based antiviral response leads to a translational arrest of viral transcripts by a process that involves Argonaute 4 (AGO4). As a result, synthesis of viral proteins is inhibited, ultimately preventing virus accumulation and spread. Whether this mechanism is generic to all *R* genes against plant-viruses remains to be investigated.

More recently, two independent studies were published that showed that the translation of *R* genes is tightly controlled through the activity of miRNAs. One study showed the miR482/2118 superfamily negatively controlled the translation of NB-LRR proteins by targeting its P-loop motif (Shivaprasad et al., 2012), while Li et al. (2012) showed that other miRNA families controlled the translation of NB-LRR proteins as well, with the TIR-NB-LRR protein N as example. During the on-going “arms race” between virus and host, viruses counter-defend against PTI/antiviral RNAi by their RSS proteins, some of which exhibit strong affinity to bind small (si- and mi-)RNAs. As a consequence such RSS proteins may suppress the miRNA induced silencing of *R* genes, and lead to enhanced expression of the *R* genes and induction of ETI. Considering that high expression levels of *R* genes lead to auto-immunity (Xia et al., 2013), viral RSS proteins with affinity to sRNAs thus may play a major role in the induction of HR. However, viral RSS are clearly not the only criterium as observed by the virus-specific activation of *R* genes and final HR, which indicates that a more complex interplay between viral effectors and *R* gene products is (additionally) required.

## Downstream defense responses

Dominant *R* genes trigger a hypersensitive response (HR) or an extreme response (ER) in case the reaction occurs in a single cell. Both involve a programmed cell death (PCD) response that rapidly kills infected cells and prevents systemic spread of the (virus) pathogen. An induced HR is quite characteristic and involves the activation and expression of SA, jasmonic acid (JA), nitride oxide (NO), ethylene, reactive oxygen species (ROS), and Ca^2+^, and expression of Pathogenesis Related (*PR*)-genes. While each component has a specificity toward certain pathogens, only SA, ROS, and Ca^2+^ seem to be effective against viruses (Loebenstein, 2009; Carr et al., 2010).

In the past, an HR was considered to be part of the resistance response, however, recent insights into R protein downstream signaling indicate that programmed cell death (HR) and resistance are distinct physiological pathways (Bendahmane et al., 1999; Bai et al., 2012). One of the best examples in support of this comes from studies on Rx-based resistance against PVX. The *Rx* gene product is a CC-NB-LRR protein from potato that is triggered by the PVX structural CP protein. The Rx protein localizes in the cytoplasm while shuttling to and from the nucleus thereby triggering resistance (Slootweg et al., 2010). Although an HR is monitored, this response can be knocked out without affecting Rx-mediated resistance against PVX (Bendahmane et al., 1999). Another example is the *N*-gene mediated resistance against TMV as described above in section Model of *R* Gene Recognition (Bhattacharjee et al., 2009). Similar observations have been made by others (Cole et al., 2001; Cawly et al., 2005; Genger et al., 2008; Bulgarelli et al., 2010; Bai et al., 2012) and indicate that the actual resistance response is different from an HR, although both mostly are triggered and may act in concert to clear viral invasions. Whether both are triggered by a pathogens' Avr determinant or whether HR is sequentially triggered following the *R* gene response is not clear.

While several interacting proteins have been identified that control R protein activity in the absence of pathogens (e.g., RAR1, SGT1, WRKY1, TPR1, Hsp90), more recently it has been found that there are also proteins that modulate the strength of defense responses (RanGAP, EDS1-PAD4) (Lu et al., 2003; Wiermer et al., 2005; Sacco et al., 2009). The benefit for the plant in a modulated fine-tuning of the ETI response to specific pathogens lies in improved effector sensing and minimizing the fitness costs involved with certain defense responses (free radical production, defense protein synthesis, cell death) (Padmanabhan and Dinesh-Kumar, 2010). While *R* gene mediated defense is taking place locally at the site of entry, it is also able to induce defense signaling responses in distally located tissues, known as systemic acquired resistance (SAR) (Vlot et al., 2008). For both the *N* gene in tobacco and *Rx1* in potato, SAR has been demonstrated (Delaney et al., 1994; Liu et al., 2010) and in both cases this response is mediated by the SA-dependent pathway as a mobile signal. SAR also prevents infection by other pathogens in the host by activating *PR* genes in the systemic tissue, which are used as a hallmark of SAR and were shown to have antimicrobial activity, although a direct inhibition on virus replication has not been shown (Durrant and Dong, 2004; Loebenstein, 2009; Carr et al., 2010).

## Functional and structural homology of plant- and animal-sensors of innate immunity

**Figure 2:**
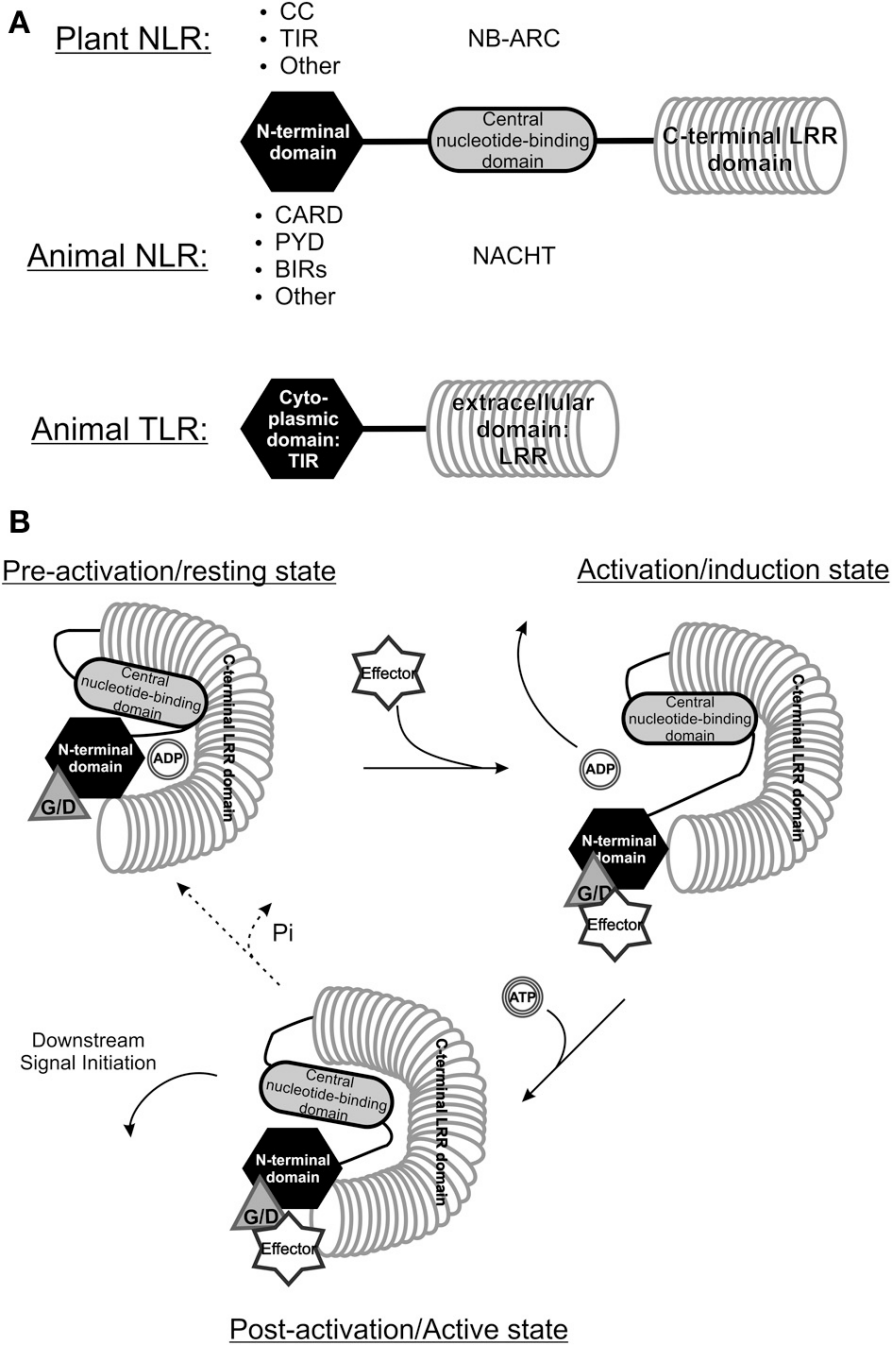
**Comparison between the structure of plant and animal NLRs. (A)** The structure of “Nucleotide binding and leucine rich repeat proteins” (NLRs) from the animal and plant kingdom share highest homology, as all proteins belonging to this class have a C-terminal leucine rich repeat (LRR), a central nucleotide binding domain and a varying N-terminal domain (modified from Maekawa et al., 2011b). Animal TLRs also contain an (extracellular) LRR domain and possess a TIR-domain, they do however, lack a nucleotide binding domain. CC, Coiled-coil; TIR, Toll-interleukin receptor; CARD, Caspase-activation and recruitment domain; PYR, Pyrin domain; BIR, Baculovirus inhibitor-of-apoptosis repeats; NB-ARC, Nucleotide binding and Apaf1-R protein-CED4 domain; NACHT, NAIP – CIITA - HET-E - TP1 domain. **(B)** A model of NB-LRR R protein recognizing a specific Avr protein through a guardee or decoy host protein. Upon interaction with the Avr protein the R protein conformationally changes and the ADP can be exchanged for ATP, leading to a second conformational change triggering downstream resistance (Modified from Lukasik and Takken, 2009). Whether the R protein returns to its resting state is not known yet. G/D, Guardee/Decoy.

Viruses are pathogens to many different organisms and, irrespective of the host species they infect, often share similarities in genome organization and functions of encoded proteins. A good example of this is exemplified by viruses from the *Bunyaviridae* family where all members infect animals with the exception of those from the *Tospovirus* genus that, besides infecting their thrips vector, are plant pathogenic and are postulated to have evolved from a common ancestor. Likewise, as a result of co-evolution driven by host-pathogen interactions, plants and animals show some similarities in their innate immune sensory systems. While in plants the aforementioned *R* genes are important in mounting an ETI response, in animals two major classes are distinguished that (partially) share similarity to these *R* genes, however both function as PRRs in the PTI response. The first major class present the “nucleotide-binding domain and leucine-rich repeat”-proteins (NLRs) and the second class is that of Toll like receptors (TLRs), which are all found to function as PRRs in the PTI response. Both are immune receptors aimed at detecting “foreign” structures and activating downstream defense responses. The family of NLRs share the most homology, as evidenced when looking at *R* genes from plants and NACHT-LRR encoding genes from the animal kingdom (NAIP–CIITA–HET-E–TP1 domain) (Leipe et al., 2004; Takken et al., 2006; Maekawa et al., 2011b). They both contain a nucleotide binding domain and a LRR (Maekawa et al., 2011b) (Figure 2). Additionally, plant R proteins also share homology at their N-termini with animal TLRs, membrane-bound immune receptors that function as sensors in pathogen recognition across membranes.

The NLRs of both plant and animal kingdom share homology through the presence of the Leucine-rich repeats (LRR) in these proteins. The most prevalent type of R proteins in plants belong to the NB-LRR protein structural class, from which the central nuclear binding domain (NBS) exhibits similarity to the nucleotide binding domain in several metazoan apoptosis regulating proteins like Apaf-1 from mammals and CED-4 from *C. elegans*. Due to the latter the NBS domain is also often referred to as NB-ARC domain (from Apaf1–R-protein–CED4) (Van Der Biezen and Jones, 1998; Takken et al., 2006). The N-terminal domain furthermore separates different classes of *R* genes; TIR-NB-LRRs harbor a Toll/Interleukin-1 Receptor domain with similarity to metazoan TLRs (Burch-Smith et al., 2007; Bernoux et al., 2011; Maekawa et al., 2011b; Hao et al., 2013). CC-NB-LRRs contain coiled coil domain forming the more irregular shaped intertwined alpha-helices (Lupas, 1997). Parallel to the discovery of many NB-LRR encoding *R* genes in plants in the recent years, the search for homology to Apaf-1 and CED-4 resulted in the recognition of the NACHT-LRR protein family in vertebrates (Koonin and Aravind, 2000; Leipe et al., 2004). Animal NLRs activate caspase-1 leading to activation and release of the cytokine interleukin-1 beta (Case, 2011), which subsequently induces local and systemic immune reactions. Similar to plant NB-LRR proteins, NLRs were found to act as higher-order active complexes, e.g., NLRP1-3 and NLRC4 form a complex often termed the inflammasome (Maekawa et al., 2011b).

TLRs represent the best studied family of PRRs in mammals so far. They are transmembrane glycoprotein receptors with an extracellular PAMP-binding domain consisting of multiple LRR that fold into a “horseshoe” structure. Additionally, it possesses intracellular signaling regions that have similarity to the intracellular domain of the Interleukin-I receptor ((IL-1R), which is referred to as Toll/IL-1R (TIR) domain that mediates downstream signaling upon activation of the receptor. TLRs initiate signal cascades involving the activation of nuclear factor kappa b (NF-κ B), mitogen-activated protein kinase (MAPK), and interferon regulatory factors (IRFs). This subsequently leads to a concerted expression of interferons, cytokines, and chemokines. Finally, inflammatory processes, cell cycle arrest, and cell death are induced (Honda et al., 2005; Kaisho and Akira, 2006). In humans, 10 TLRs have been identified of which TLR2, -3, -4, -7, and -8 are involved in sensing structural components of RNA viruses like double-stranded RNA, single-stranded RNA and viral glycoproteins (Bowie and Unterholzner, 2008). While most TLRs are involved in extracellular recognition of PAMPs, TLR3, -7, and -8 are primarily restricted to intracellular compartments (endoplasmic-reticulum (ER), endosomes etc.) where they sense structural components of viral RNA. Besides TLRs cytosolic retinoic acid-inducible gene I (RIG-I)-like receptors (RLRs; RIG-I, MDA5, and LSP2) have been identified as sensors of RNA viruses and are involved in the very early response of some RNA viruses (Bowie and Unterholzner, 2008; Gerlier and Lyles, 2011; Jensen and Thomsen, 2012).

Pathogen recognition in both animal and plant kingdoms involves the LRR domain, which binds the ligand in its horseshoe shape, often followed by activation of a signaling cascade through kinase phosphorylation. Structural similarities between animal TLR/NLR and plant NB-LRR proteins point to a convergent evolution of these defense-related pathways (Yue et al., 2012). However innate immunity in animals and plants differs substantially in their downstream defense response, with interleukin/interferon-activated inflammatory responses combined with activating the adaptive immune system in mammalian systems and a resistance response (as explained before) often seen as a programmed cell death response in form of HR in plants.

## Outlook

Dominant resistance against plant viruses are of increasing interest to breeders and scientists in the past years. More and more is known about the molecular mechanisms behind *R* gene mediated defense and the induction of the HR. However, the number of well-studied examples is still very limited, and therefore makes it difficult to extrapolate to other less studied *R* genes. In depth studies on other *R* genes from different crops providing resistance against different phytopathogens will have to show whether there is common mechanism of defense shared between all *R* genes, or whether specialization between different classes of *R* genes occurs. Knowledge on mammalian innate immunity sensors and their mode of action may provide interesting and cross pollinating views in this. The rapid generation of resistance breaking virus isolates against dominant resistance genes already indicates the importance for alternative resistance genes, to provide more durable, and effective resistances.

### Conflict of interest statement

The authors declare that the research was conducted in the absence of any commercial or financial relationships that could be construed as a potential conflict of interest.
