# Supplementary material for: Dominant resistance against plant viruses
Source: Front Plant Sci. 2014 Jun 27;5:307. doi: 10.3389/fpls.2014.00307 (PMC4073217; doi:10.3389/fpls.2014.00307)
Supplement: Supplementary file 1 [file DataSheet1.PDF]

Frontiers in Plant-Microbe Interactions

Dominant resistance against plant viruses: Supplementary Material

Dryas de Ronde[1], Patrick Butterbach[1] and Richard Kormelink[1*]

[1]Laboratory of Virology, Department of Plant Sciences, Wageningen University, Wageningen, The Netherlands.
Tel.: +31-(0)317-483090
Fax: +31-(0)317-484820

Correspondence:
Richard Kormelink: Richard.kormelink@wur.nl
Laboratory of Virology, Department of Plant Sciences, Wageningen University, Droevendaalsesteeg 1, 6708 PB Wageningen, The Netherlands.

Table S1.1. Complete overview of dominant resistances known against plant viruses, and listed in alphabetical order based on the host of origin.

Plant host	*R* gene	Cloned/type	Recognises	Virus genus	AVR	Reference
Arabidopsis thaliana<br>Mouse ear cress	*HRT*	Yes: CC-NB-LRR<br>[HR]	TCV [*Turnip crinkle virus*]	*Carmovirus*	CP	1, 2
	JAX1	Yes: Jacalin-like<br>[lectin gene]	*Broad resistance against potexvirus*	*Potexvirus*	unknown	287
	RCY1	Yes: CC-NB-LRR<br>[HR]	CMV [*Cucumber mosaic virus*]	*Cucumovirus*	CP	3-6
	RTM1<br>*RTM2*<br>*RTM3*	Yes: Jacalin-like<br>[prev. Syst. Mov.]<br>[RTM3 not cloned]	TEV [*Tobacco etch virus*]<br>PPV [*Plum pox virus*]<br>LMV [*Lettuce mosaic virus*]	*Potyvirus*	CP<br>CP<br>CP	7-9
	TuNI	No [syst. HR]	TuMV [*Turnip mosaic virus*]	*Potyvirus*	P3	10, 11
Beta vulgaris<br>Common beet	*Bm*	No	BtMV [*Beet mosaic virus*]	*Potyvirus*	unknown	12
	Rz1<br>*Rz2*<br>*Rz3*	No [partial resistance]	BNYVV [*Beet necrotic yellow vein virus*]	*Benyvirus*	unknown	13, 14
Brachypodium distachyon<br>Purple false brome	*Bsr1*	No<br>'CC-NB-LRR'	BSMV [*Barley stripe mosaic virus*]	*Hordeivirus*	TGB1 (MP)	15, 16
Brassica campestris<br>Field mustard	*BcTuR3*	Yes:<br>TIR-NB-LRR	TuMV [*Turnip mosaic virus*]	*Potyvirus*	unknown	17, 18
	Unknown gene	No			unknown	17
Brassica napus<br>Rapeseed	*TuRB01,*<br>*TuRB01b,*<br>*TuRB02,*<br>*TuRB03,*<br>*TuRB04,*<br>*TuRB05*	No [ER]<br>No<br>No [Partial Res.]<br>No [ER]<br>No [ER]<br>No [HR]	TuMV [*Turnip mosaic virus*]	*Potyvirus*	TuRB01: CI<br>TuRB01b: CI<br>Unknown<br>TuRB03: P3<br>TuRB04: P3<br>TuRB05: CI	19-22
	Tum	No	TuMV [*Turnip mosaic virus*]	*Potyvirus*	Unknown	17
	Monogenic	No[ER]	TuMV [*Turnip mosaic virus*]	*Potyvirus*	Unknown	
Brassica rapa<br>Turnip	*TuMV-R*	No<br>'CC-NB-LRR'	TuMV [*Turnip mosaic virus*]	*Potyvirus*	Unknown	290
	TuRB01b	No [ER]	TuMV [*Turnip mosaic virus*]	*Potyvirus*	CI	19, 22-24
	TuRB07	No<br>'CC-NB-LRR'	TuMV [*Turnip mosaic virus*]	*Potyvirus*	unknown	289

Capsicum annuum<br>Pepper	*Pvr4*<br>(locus)	No [ER]	PVY [*Potato virus Y*]<br>PepMoV [*Pepper mottle virus*]<br>PepYMV [*Pepper yellow mosaic virus*]<br>PepSMV [*Pepper severe mosaic virus*]<br>ERV [*Ecuadorian rocoto virus*]<br>PTV [*Peru tomato mosaic virus*]	*Potyvirus*	Nlb (RdRp)	25-30
	L^{1} (locus)<br>$L^{1a\$}$ *[temp insens.]*<br>(locus)	Yes:<br>CC-NB-LRR	TMV [*Tobacco mosaic virus*]<br>ToMV [*Tomato mosaic virus*]<br>TMGMV [*Tobacco mild green mosaic virus*]<br>BPeMV [*Bell pepper mottle virus*]	*Tobamovirus*	CP<br>CP<br>CP<br>CP	25, 31-34
	Cmr1	No [blocks syst. Mov.]	CMV [*Cucumber mosaic virus*]	*Cucumovirus*	Helicase (1a)	25, 30, 35-39
	HK	No [Incomp. Dom.]	PaMMV [*Paprika mild mottle virus*]	*Tobamovirus*	Methyl transferase	40, 41
Capsicum chacoense<br>Pepper	L^{4}<br>(locus)	Yes:<br>CC-NB-LRR	TMV [*Tobacco mosaic virus*]<br>ToMV [*Tomato mosaic virus*]<br>TMGMV [*Tobacco mild green mosaic virus*]<br>BPeMV [*Bell pepper mottle virus*]<br>PaMMV [*Paprika mild mottle virus*]<br>ObPV [*Obuda pepper virus*]<br>PMMoV [*Pepper mild mottle virus*]	*Tobamovirus*	CP<br>CP<br>CP<br>CP<br>CP<br>CP<br>CP	25, 31, 32
Capsicum chinense<br>Pepper	*Tsw*	No: [HR]	TSWV [*Tomato spotted wilt virus*]	*Tospovirus*	NSs	42, 285
	L^{3}<br>(locus)	Yes:<br>CC-NB-LRR	TMV [*Tobacco mosaic virus*]<br>ToMV [*Tomato mosaic virus*]<br>TMGMV [*Tobacco mild green mosaic virus*]<br>BPeMV [*Bell pepper mottle virus*]<br>PaMMV [*Paprika mild mottle virus*]<br>ObPV [*Obuda pepper virus*]<br>PMMoV [*Pepper mild mottle virus*]	*Tobamovirus*	CP<br>CP<br>CP<br>CP<br>CP<br>CP<br>CP	31, 32,<br>43, 44
	Pvr7	No	PepMoV [*Pepper mottle virus*]	*Potyvirus*	Unknown	30
Capsicum frutescens<br>Pepper	L^{2}<br>(locus)	Yes:<br>CC-NB-LRR	TMV [*Tobacco mosaic virus*]<br>ToMV [*Tomato mosaic virus*]<br>TMGMV [*Tobacco mild green mosaic virus*]<br>BPeMV [*Bell pepper mottle virus*]<br>PaMMV [*Paprika mild mottle virus*]<br>ObPV [*Obuda pepper virus*]	*Tobamovirus*	CP<br>CP<br>CP<br>CP<br>CP<br>CP	25, 31, 32,<br>44, 45
Chenopodium amaranticolor<br>Goosefoot	Unknown	No	CaMV [*Cauliflower mosaic virus*]	*Caulimovirus*	Gene VI product	46, 47
Cucurbita moschata<br>Squash, Pumpkin	*Cmv*	No	CMV [*Cucumber mosaic virus*]	*Cucumovirus*	Unknown	48
	Wmv	No [No sympt.]	MWMV [*Watermelon mosaic virus*]	*Potyvirus*	Unknown	48, 49
	Zym	No [No sympt]	ZYMV [*Zucchini yellow mosaic virus*]	*Potyvirus*	Unknown	48, 50-53
	Slc	No	SLCV [*Squash leaf curl virus*]	*Begomovirus*	RNAs 2 and 3	54-56

Cucumis melo Muskmelon	*Mnr1*, *Mnr2*	No [cell to cell move.]	MNSV [*Melon necrotic spot virus*]	*Carmovirus*	Unknown	57
	Pvr1, *Pvr2*	Yes: TIR-NB-LRR	PRSV [*Papaya ringspot virus*]	*Potyvirus*	Unknown Unknown	50, 288
	Monogenic polygenic	No [poly: partial res]	CMV [*Cucumber mosaic virus*]	*Cucumovirus*	Unknown Unknown	58, 59
	Zym	No [No sympt]	ZYMV [*Zucchini yellow mosaic virus*]	*Potyvirus*	CP	50-53
	Wmr	No	MWMV [*Watermelon mosaic virus*]	*Potyvirus*	Unknown	50, 57, 60
	Wmv-1, *Wmv-1*[2]	No	MWMV [*Watermelon mosaic virus*]	*Potyvirus*	Unknown Unknown	61
Cucumis sativus Garden cucumber	*Wmv1-1*, *Prsv-2*	No [tol., no sympt, high titres]	PRSV [*Papaya ringspot virus*]	*Potyvirus*	Unknown Unknown	62, 63
Dioscorea rotundata White yam	*Ymv-1* (locus)	No [resistance]	YMV [*Yam mosaic virus*]	*Potyvirus*	Unknown	64
Glycine max Soybean	*Rsv1* (locus)	Yes: CC-NB-LRR [ER/HR]	SMV [*Soybean mosaic virus*]	*Potyvirus*	P3+ HC-Pro	65-69
	Rsv3 (sim Rsv1)<br><br>*Rsv4* (not sim.)	No [ER/HR/stem-tip necrosis] 'CC-NB-LRR'<br>No [broad resistance: delay repl + move.]	SMV [*Soybean mosaic virus*]	*Potyvirus*	CI<br><br>P3	68, 70-74
	Rcv	No [HR]	CCMV [*Cowpea chlorotic mottle virus*]	*Bromovirus*	unknown	75
	Rpv1, *Prmv*	No No	PeMoV [*Peanut mottle virus*]	*Potyvirus*	Unknown unknown	76, 77
	Rav1	No	AMV [*Alfalfa mosaic virus*]	*Alfamovirus*	Unknown	78
	Unknown	No	TSV [*Tobacco streak virus*]	*Ilarvirus*	Unknown	79, 80
Hordeum bulbosum Bulbous barley	*Rym14*, *Rym16* *Rym17*	No	BaYMV [*Barley yellow mosaic virus*]	*Bymovirus*	Unknown	81-85
Hordeum vulgare Barley	*Rrs-1*	No [Replication]	BSMV [*Barley stripe mosaic virus*]	*Hordeivirus*	unknown	86, 87
	Ryd2/Yd2, *Ryd3*	No [Yd2: tol. Ryd3: resist.]	BYDV [*Barley yellow dwarf virus*]	*Luteovirus*	Unknown	88-97
Lactuca saligna Willowleaf lettuce	*Rsv*, *Rsv2*	No [syst. res.]	CMV [*Cucumber mosaic virus*]	*Cucumovirus*	Unknown	98, 99
Lactuca sativa Garden lettuce	*Tu*	No	TuMV [*Turnip mosaic virus*]	*Potyvirus*	Unknown	100, 101
	Tvr1	No [partial resist.]	LNSV [*Lettuce necrotic stunt virus*]	*Tombusvirus*	Unknown	102, 103
Nicotiana edwardsonii	Unknown	No	CaMV [*Cauliflower mosaic virus*]	*Caulimovirus*	P6	104

Nicotiana glutinosa<br>Tobacco	*N*	Yes:<br>TIR-NB-LRR<br>[cell-cell mov.]	TMV [*Tobacco mosaic virus*]	*Tobamovirus*	p50 [Helicase]	105-111
	Unknown	No	CMV [*Cucumber mosaic virus*]	*Cucumovirus*	1a	112, 113
Nicotiana sylvestris<br>Wood tobacco	*N'*	No [HR]<br>'NB-LRR'	TMV [*Tobacco mosaic virus*]	*Tobamovirus*	CP	114-116
Nicotiana tabacum<br>Common tobacco	Unknown	No	CMV [*Cucumber mosaic virus*]	*Cucumovirus*	1a	112, 113, 117
	Unknown	No	ToLCNDV [*Tomato leaf curl New Delhi virus*]	*Begomovirus*	NSP<br>(BV1)	118, 119
	Unknown	No [HR]	PaLCuV [*Papaya leaf curl virus*]<br>CLCuKV [*Cotton leaf curl Kokhran virus*]	*Begomovirus*	V2	120
	Unknown	No	TAV [*Tomato aspermy virus*]	*Cucumovirus*	2b	121, 122
Nicotiana spec.	Polygenic	No [HR]	TBSV [*Tomato bushy stunt virus*]<br>CymRSV [*Cymbidium ringspot virus*]<br>CNV [*Cucumber necrosis virus*]	*Tombusvirus*	P19/P22/P41<br>P19/P22<br>P20/P21	123, 124
Pisum sativum<br>Pea	*En*	No	PEMV [*Pea enation mosaic virus*]	*Enamovirus*	Unknown	125, 126
Phaseolus vulgaris<br>Kidney bean	*I*<br>(locus)	Yes:<br>TIR-NB-LRR<br>[ER/HR/phloem necr.]	BCMV [*Bean common mosaic virus*]<br>BNMV [*Bean necrotic mosaic virus*]<br>BICMV *[Blackeye cowpea mosaic virus]*<br>AzMV *[Azuki mosaic virus]*<br>CABMV [*Cowpea aphid-borne mosaic virus*]<br>PWV [*Passionfruit woodiness virus*]<br>SMV [*Soybean mosaic virus*]<br>ThPV *[Thailand passiflora virus]*<br>WMV [*Watermelon mosaic virus*]<br>ZYMV [*Zucchini yellow mosaic virus*]	*Potyvirus*	unknown	127-133
	PvVTT1	Yes:<br>TIR-NB-LRR<br>[HR]	BDMV [*Bean dwarf mosaic virus*]	*Begomovirus*	BV1<br>(NSP)	134-139
	Amv<br>*Amv-2*	No	AMV [*Alfalfa mosaic virus*]	*Alfamovirus*	Unknown	140, 141
	Bgp-1	No [Norm. pod form.]	BGMV [*Bean golden mosaic virus*]	*Begomovirus*	Unknown	142
	Bgp-2	No			Unknown	143, 144
	By,<br>*By-2*	No	BYMV [*Bean yellow mosaic virus*]	*Potyvirus*	Unknown	145, 146
	Bcm	No	BICMV *[Blackeye cowpea mosaic virus*]	*Potyvirus*	Unknown	140, 147
	Monogenic	No	BBWV [*Broad bean wilt virus*]	*Fabavirus*	Unknown	148
	Cam<br>*Cam2*	No	CABMV [*Cowpea aphid-borne mosaic virus*]	*Potyvirus*	Unknown	149, 150

	Pwv	No [Syst. res.]	PWV [*Passion fruit woodiness virus*]	*Potyvirus*	Unknown	151
	Smv<br><br>*Hss*	No [Syst. res.]<br><br>No	SMV [*Soybean mosaic virus*]	*Potyvirus*	Unknown<br><br>Unknown	152, 153
	Wmv<br><br>*Hsw*	No [syst. spread]<br><br>No [full resis.]	WMV [*Watermelon mosaic virus*]	*Potyvirus*	Unknown<br><br>Unknown	154, 155
	PvCMR1<br>(*RT4-4*)	Yes:<br>TIR-NB-LRR<br>[syst. necrosis]	CMV [*Cucumber mosaic virus*]	*Cucumovirus*	2a	156
	Bct	No	BCTV [*Beet curly top virus*]	*Curtovirus*	Unknown	157
	Azm1<br>*Azm2*	No	AzMV *[Azuki mosaic virus]*	*Potyvirus*	Unknown	133
Poncirus trifoliate<br>Trifoliate orange	*Ctv*<br>(locus)	Yes:<br>CC-NB-LRR	CTV [*Citrus tristeza virus*]	*Closterovirus*	unknown	158-160
Rubus idaeus<br>Raspberry	*Bu*	No	RBDV [*Raspberry bushy dwarf virus*]	*Idaeovirus*	Unknown	161-164
	2 genes	No	RcRSV [*Raspberry ringspot virus*]	*Nepovirus*	Unknown	165
	2 genes	No	TBRV [*Tomato black ring virus*]	*Nepovirus*	Unknown	165
Saccharum spontaneum<br>Wild sugarcane	Monogenic	No	SCMV [*Sugarcane mosaic virus*]	*Potyvirus*	Unknown	17
Solanum chilense<br>Tomato	*Ty-1*<br>*Ty-3*	Yes:<br>RDR [Tol.]	TYLCV [*Tomato yellow leaf curl virus*]	*Begomovirus*	No	30, 166, 167
Solanum esculentum<br>Tomato	Unknown	No	ToLCNDV [*Tomato leaf curl New Delhi virus*]	*Begomovirus*	NSP	118, 119
	Unknown	No	ToLCJV [*Tomato leaf curl Java virus*]		V2	119
Solanum habrochaites<br>Tomato	*Ty-2*	No [Tol.]	TYLCV [*Tomato yellow leaf curl virus*]	*Begomovirus*	Unknown	167
Solanum hirsutum<br>Tomato	Monogenic	No [Tol.]	PTV [*Peru tomato mosaic virus*]	*Potyvirus*	Unknown	168
	Tm-1	Yes:<br>TIM-barrel-like domain protein<br>[ER]<br>[Replication]	ToMV *[Tomato mosaic virus*]	*Tobamovirus*	Replicase:<br>Helicase-domain	169-174
Solanum lycopersicum<br>Tomato	*Am*	No [ER]	AMV [*Alfalfa mosaic virus*]	*Alfamovirus*	Unknown	175
	Unknown	No [ER: immune]	PVY [*Potato virus Y*]	*Potyvirus*	Unknown	176, 177
	Cmr	No	CMV [*Cucumber mosaic virus*]	*Cucumovirus*	Unknown	178
Solanum peruvianum<br>Tomato	*Sw5b*	Yes:<br>CC-NB-LRR [HR]	TSWV [*Tomato spotted wilt virus*]<br>and other tospoviruses	*Tospovirus*	NSm	179-183, 286
	Unknown	No [ER]	TYTV [*Tomato yellow top virus*]	*Luteovirus*	Unknown	184-187

	Tm-2	Yes:<br>CC-NB-LRR<br>[HR]	TMV [*Tobacco mosaic virus*]<br>ToMV [*Tomato mosaic virus*]<br>and other tobamoviruses	*Tobamovirus*	30kD MP.	171, 188, 189
	$Tm\text{-}2^2$	Yes:<br>CC-NB-LRR<br>[HR]	ToMV [*Tomato mosaic virus*]<br>TMV [*Tobacco mosaic virus*]<br>and other tobamoviruses	*Tobamovirus*	30kD MP.	171, 190-193
Solanum acaule<br>Potato	Rx_{acl}	No [ER]	PVX [*Potato virus X*]	*Potexvirus*	Unknown	194-196
	X^I	No [ER]	PVX [*Potato virus X*]	*Potexvirus*	Unknown	194-196
	$Rx_{acl}{}^{n}$<br>($X^n = Nx_{acl}$)	No [HR]	PVX [*Potato virus X*]	*Potexvirus*	Unknown	194-196
Solanum chacoense<br>Potato	Ny_{chc}	No [HR]	PVY [*Potato virus Y*]<br>PVA [*Potato virus A*]	*Potyvirus*	Unknown	194, 195
Solanum demissum<br>Potato	Ny_{dms}<br>(= N^y)	No [ER]	PVY [*Potato virus Y*]<br>PVA [*Potato virus A*]	*Potyvirus*	Unknown	195-199
	$Ry_{dms}{}^{a}$<br>(Na_{dms}=N^a)	No [HR]	PVA [*Potato virus A*]		Unknown	
Solanum etuberosum<br>Potato	Rlr_{etb}	No	PLRV [*Potato leafroll virus*]	*Polerovirus*	Unknown	196, 200-203
Solanum gourlayi<br>Potato	*Gm*	No	PVM [*Potato virus M*]	*Carlavirus*	Unknown	196, 204, 205
Solanum hougasii<br>Potato	Ry_{hou}	No [ER]	PVY [*Potato virus Y*]<br>PVA [*Potato virus A*]	*Potyvirus*	Unknown	195, 198, 199
Solanum megistracrolobum<br>Potato	*Nm*	No [HR]	PVM [*Potato virus M*]	*Carlavirus*	Unknown	196, 205-208
	Rm	No [HR]			Unknown	
Solanum sparsipilum<br>Potato	Nc_{spl}	No [HR]	PVY [*Potato virus Y*]	*Potyvirus*	HcPro	196, 209
	$Nx_{tbr}{}^{spl}$	No [HR]	PVX [*Potato virus X*]	*Potexvirus*	Unknown	195, 196
Solanum stoloniferum<br>Potato	Ry_{sto} (Ry=R^1)	No [ER]<br>'NB-LRR'	PVA [*Potato virus A*]	*Potyvirus*	Unknown	30, 195, 196 198, 199, 210-213
			PVV [*Potato virus V*]		Unknown	
			PVY [*Potato virus Y*]		NlaPro	
	$Ry_{sto}{}^{na}$ (=R^2)	No [HR]	PVA [*Potato virus A*]	*Potyvirus*	Unknown	195, 196, 214, 215
		No [HR/ER]	PVV [*Potato virus V*]			
		No [ER]	PVY [*Potato virus Y*]			
	$Ry_{sto}{}^{rna}$<br>(= R^3)	No [HR/ER]	PVA [*Potato virus A*]<br>PVY [*Potato virus Y*]	*Potyvirus*	Unknown	195, 196
	$Ry_{sto}{}^{n1}$ (Ryn = $Ny_{sto}{}^{1}$)	No [HR]	PVY [*Potato virus Y*]	*Potyvirus*	Unknown	194-196

	Ry_{sto}^{n2} (*R^5= Ny_{sto}^2*)	No [ER]	PVY [*Potato virus Y*]	*Potyvirus*	Unknown	195, 196
	Na_{sto} (*R^6= Rym*)	No [HR]	PVA [*Potato virus A*]	*Potyvirus*	Unknown	194-196
	Ra (=*Ra_{sto}*)	No [HR]	PVA [*Potato virus A*]	*Potyvirus*	Unknown	196, 216
Solanum tuberosum Potato	*Na_{KE}* (=*Na_{KEtbr}*)	No [HR]	PVA [*Potato virus A*]	*Potyvirus*	unknown	196, 217
	Na_{tbr} (=*Na*)	No [HR]	PVA [*Potato virus A*] PVY [*Potato virus Y*]	*Potyvirus*	Unknown	195, 196, 198, 199, 218
	Nb (=*Nb_{tbr}*)	No [HR]	PVX [*Potato virus X*]	*Potexvirus*	25K	195, 196, 219, 220
	Nc_{tbr} (=*Nc*)	No [HR]	PVY [*Potato virus Y*]	*Potyvirus*	HcPro	195, 196, 209, 220
	Ns	No [HR]	PVS [*Potato virus S*]	*Carlavirus*	Unknown	196, 204, 206, 221-223
	Nv_{tbr} (=*Nv*)	No [HR]	PVV [*Potato virus V*]	*Potyvirus*	unknown	196, 198, 199, 214, 224
	Nx (=*Nx_{tbr}*)	No [HR]	PVX [*Potato virus X*]	*Potexvirus*	CP	195, 196, 198, 199, 225
	Ny_{adg}	No [HR]	PVY [*Potato virus Y*]	*Potyvirus*	Unknown	196, 226
	Ny_{tbr} (=*Ny*)	No [HR]	PVY [*Potato virus Y*]	*Potyvirus*	HcPro	195, 196, 198, 199, 209, 227
	Ny-1	No [HR]	PVY [*Potato virus Y*]	*Potyvirus*	Unknown	196, 228
	Ra_{adg}	No [ER]/[HR]	PVA [*Potato virus A*]	*Potyvirus*	Unknown	196, 229
	Rx1	Yes: CC-NB-LRR [ER/HR]	PVX [*Potato virus X*] and other potex viruses	*Potexvirus*	CP	195, 198, 199, 230-234
	Rx2	Yes: CC-NB-LRR	PVX [*Potato virus X*]	*Potexvirus*	CP	138, 232
	Ry_{adg}	No [No sympt/ER]	PVY [*Potato virus Y*]	*Potyvirus*	NIa protease	196, 198, 199, 213, 226, 235
	ss_{tbr} (=*s*)	No [ER]	PVS [*Potato virus S*]	*Carlavirus*	Unknown	196, 236
	Y-1	Yes: TIR-NB-LRR.	PVY [*Potato virus Y*]	*Potyvirus*	unknown	237, 238
	Polygenic	No [ER/HR]	TRV [*Tobacco rattle virus*]	*Tobravirus*	MP [29K]	239

Sorghum bicolor<br>Sorghum	*'Krish'*	No	MDMV [*Maize dwarf mosaic virus*]<br>JGMV [*Johnsongrass mosaic virus*]<br>SCMV [*Sugarcane mosaic virus*]	*Potyvirus*	Unknown	17, 240
Thinopyrum intermedium<br>Intermediate wheatgrass	*Bdv2*<br>*Bdv3*<br>*Bdv4*	No [resistance]	BYDV [*Barley yellow dwarf virus*]	*Luteovirus*	unknown	241-247
	Wsm1	No	WSMV [*Wheat streak mosaic virus*]	*Tritimovirus*	unknown	248-250
Triticum aestivum<br>Common wheat	*Bdv1*	No [Tol.]	BYDV [*Barley yellow dwarf virus*]	*Luteovirus*	unknown	247, 251
	Wss1	No	WSSMV [*Wheat spindle streak mosaic virus*]	*Bymovirus*	Unknown	252, 253
	Sbm<br>[locus]	No	SBWMV [*Soil-borne wheat mosaic virus*]<br>SBCMV [*Soil-borne cereal mosaic virus*]	*Furovirus*	Unknown	254, 255
Vicia faba<br>Broad bean	*Bym-1,*<br>*Bym-2*	No	BYMV [*Bean yellow mosaic virus*]	*Potyvirus*	Unknown	17
Vigna mungo<br>Black gram	*CYR1*	Yes:<br>CC-NB-LRR	MYMV [*Mungbean yellow mosaic virus*]	*Begomovirus*	CP	256, 257
Vigna unguiculata<br>Cowpea	Monogenic	No [ER]	CCMV [*Cowpea chlorotic mottle virus*]	*Bromovirus*	Unknown	258, 259
	Cry	No [HR]	CMV [*Cucumber mosaic virus*]	*Cucomovirus*	2a polymerase	260-265
	Monogenic	No [partial Dom/ER]	SBMV [*Southern bean mosaic virus*]	*Sobemovirus*	Unknown	266, 267
	Monogenic	No	TRSV [*Tobacco ringspot virus*]	*Nepovirus*	Unknown	259, 268, 269
	Monogenic	No	CABMV [*Cowpea aphid-borne mosaic virus*]	*Potyvirus*	Unknown	150,270, 271
	Cpa	No [ER]	CPMV [*Cowpea mosaic virus*]	*Comovirus*	Protease,<br>24K Pro	272, 273
Zea mays<br>Maize/corn	*Mdm-1 (Rdm-1)*	No	MDMV [*Maize dwarf mosaic virus*]	*Potyvirus*	Unknown	17, 274
	Msv1	No [Tol./Partial Dom]	MSV [*Maize streak virus*]	*Mastrevirus*	Unknown	275, 276
	*Scmv*1 *Scmv2*<br>*Rscmv1*<br>*Rscmv2*	No (all)	SCMV [*Sugarcane mosaic virus*]	*Potyvirus*	Unknown<br>Unknown<br>Unknown<br>Unknown	243, 277-282
	Wsm1<br>*Wsm2*<br>*Wsm3*	No	WSMV [*Wheat streak mosaic virus*]<br>MDMV [*Maize dwarf mosaic virus*]<br>SCMV [*Sugarcane mosaic virus*]<br>SrMV [*Sorghum mosaic virus*]<br>JGMV [*Johnsongrass mosaic virus*]	*Tritimovirus*<br>*Potyvirus*	Unknown	17, 283, 284

1. (Cooley et al., 2000); 2. (Ren et al., 2000); 3. (Takahashi et al., 2001); 4. (Takahashi et al., 2002); 5. (Takahashi et al., 2004); 6. (Sekine et al., 2006); 7. (Chisholm et al., 2000); 8. (Whitham et al., 2000); 9. (Decroocq et al., 2009); 10. (Kaneko et al., 2004); 11. (Kim et al., 2010); 12. (Lewellen, 1973); 13. (Amiri et al., 2003); 14. (Acosta-Leal et al., 2010); 15. (Cui et al., 2012); 16. (Lee et al., 2012); 17. (Provvidenti and Hampton, 1992); 18. (Ma et al., 2010); 19. (Hughes et al., 2002); 20. (Hughes et al., 2003); 21. (Jenner et al., 2000); 22. (Walsh et al., 2002); 23. (Lehmann et al., 1997); 24. (Lim et al., 1978); 25. (Moury and Verdin, 2012); 26. (Janzac et al., 2009); 27. (Janzac et al., 2010); 28. (Caranta et al., 1999); 29. (Dogimont et al., 1996); 30. (Grube et al., 2000); 31. (Tomita et al., 2008); 32. (Tomita et al., 2011); 33. (Matsumoto et al., 2008); 34. (Sawada et al., 2004); 35. (Kang et al., 2010); 36. (Kang et al., 2012); 37. (Ben Chaim et al., 2001); 38. (Caranta et al., 2002); 39. (Nono-Womdim et al., 1991); 40. (Sawada et al., 2005); 41. (Matsumoto et al., 2009); 42. (Jahn et al., 2000); 43. (de la Cruz et al., 1997); 44. (Holmes, 1937); 45. (Berzal-Herranz et al., 1995); 46. (Kiraly et al., 1999); 47. (Schoelz et al., 1986); 48. (Brown et al., 2003); 49. (Gilbert-Albertini et al., 1993); 50. (Anagnostou et al., 2000); 51. (Danin-Poleg et al., 2002); 52. (Grumet, 1995); 53. (Pitrat and Lecoq, 1984); 54. (Paris and Brown, 2005); 55. (Montes-Garcia et al., 1998); 56. (Kang et al., 2005); 57. (Mallor et al., 2003); 58. (Lecoq et al., 1982); 59. (Pitrat and Lecoq, 1980); 60. (Gilbert et al., 1994); 61. (Pitrat and Lecoq, 1983); 62. (Grumet et al., 2000); 63. (Wai and Grumet, 1995); 64. (Mignouna et al., 2002); 65. (Hayes et al., 2004); 66. (Hajimorad and Hill, 2001); 67. (Hajimorad et al., 2005b); 68. (Wen et al., 2013); 69. (Eggenberger et al., 2008); 70. (Hayes et al., 2000); 71. (Khatabi et al., 2012); 72. (Jeong et al., 2002); 73. (Yu et al., 1994); 74. (Maroof et al., 2008); 75. (Goodrick et al., 1991); 76. (Buss et al., 1985); 77. (Boerma and Kuhn, 1976); 78. (Kopisch-Obuch et al., 2008); 79. (Hobbs et al., 2012); 80. (Wang et al., 2005); 81. (Chelkowski et al., 2003); 82. (Neuhaus et al., 2003); 83. (Ruge et al., 2003); 84. (Ruge-Wehling et al., 2006); 85. (Kai et al., 2012); 86. (Edwards and Steffenson, 1996); 87. (Zheng and Edwards, 1990); 88. (Ford et al., 1998); 89. (Chalhoub et al., 1995); 90. (Collins et al., 1996); 91. (Delogu et al., 1995); 92. (Jefferies et al., 2003); 93. (Larkin et al., 1991); 94. (Makkouk et al., 1994); 95. (Niks et al., 2004); 96. (Ovesna et al., 2000); 97. (Paltridge et al., 1998); 98. (Edwards et al., 1983); 99. (Provvidenti et al., 1980); 100. (Robbins et al., 1994); 101. (Montesclaros et al., 1997); 102. (Grube et al., 2005); 103. (Simko et al., 2009); 104. (Cawly et al., 2005); 105. (Whitham et al., 1994); 106. (Erickson et al., 1999); 107. (Baker et al., 1995); 108. (Dinesh-Kumar et al., 1995); 109. (Dinesh-Kumar et al., 2000); 110. (Padgett and Beachy, 1993); 111. (Padgett et al., 1997); 112. (Salanki et al., 2007); 113. (Diveki et al., 2004); 114. (Saito et al., 1987); 115. (Knorr and Dawson, 1988); 116. (Dardick et al., 1999); 117. (Troutman and Fulton, 1958); 118. (Hussain et al., 2005); 119. (Sharma and Ikegami, 2010); 120. (Mubin et al., 2010); 121. (Li et al., 1999); 122. (Chen et al., 2008); 123. (Angel et al., 2011); 124. (Angel and Schoelz, 2013); 125. (Weeden and Provvidenti, 1988); 126. (Yu et al., 1995); 127. (Vallejos et al., 2006); 128. (Ariyarathne et al., 1999); 129. (Collmer et al., 2000); 130. (Kelly et al., 1995); 131. (Kyle et al., 1986); 132. (Fisher and Kyle, 1994); 133. (Fisher and Kyle, 1996); 134. (Zhou et al., 2007); 135. (Garrido-Ramirez et al., 2000); 136. (Seo et al., 2004); 137. (Seo et al., 2007); 138. (Wang et al., 1999); 139. (Gururani et al., 2012); 140. (Zaumeyer and Meiners, 1975); 141. (Wade and Zaumeyer, 1940); 142. (Roman et al., 2004); 143. (Singh and Schwartz, 2010); 144. (Osorno et al., 2007); 145. (Park and Tu, 1991); 146. (Schroeder and Provvidenti, 1968); 147. (Kyle and Dickson, 1988); 148. (Provvidenti, 1988); 149. (Provvidenti et al., 1983); 150. (Bashir et al., 2002); 151. (Provvidenti, 2000); 152. (Kyle and Provvidenti, 1993); 153. (Provvidenti et al., 1982); 154. (Provvide.R, 1974); 155. (Kyle and Provvidenti, 1987); 156. (Seo et al., 2006); 157. (Miklas et al., 2009); 158. (Yang et al., 2003). 159. (Rai, 2006); 160. (Harper et al., 2010); 161. (Knight and Barbara, 1981); 162. (Taylor and Martin, 1999); 163. (Jones et al., 1982); 164. (Ward et al., 2012); 165. (Jennings, 1964); 166. (Hanson et al., 2000); 167. (Verlaan et al., 2013); 168. (Hikida and Raymer, 1972); 169. (Ishibashi et al., 2007); 170. (Ishibashi et al., 2012); 171. (Pelham, 1966); 172. (Yamafuji et al., 1991); 173. (Meshi et al., 1988); 174. (Kato et al., 2013); 175. (Parrella et al., 2004); 176. (Takacs et al., 2003); 177. (Takacs et al., 2006); 178. (Stamova and Chetelat, 2000); 179. (Finlay, 1953); 180. (Holmes, 1948); 181. (Brommonschenkel et al., 2000); 182. (Hallwass et al., Accepted for publication); 183. (Hoffmann et al., 2001); 184. (Hassan and Thomas, 1983); 185. (Hassan and Thomas, 1984a); 186. (Hassan and Thomas, 1984b); 187. (Hassan and Thomas, 1988); 188. (Hall, 1980); 189. (Meshi et al., 1989); 190. (Weber et al., 1993); 191. (Lanfermeijer et al., 2003); 192. (Lanfermeijer et al., 2005); 193. (Tanksley et al., 1998); 194. (Ross, 1961); 195. (Cockerham, 1970); 196. (Palukaitis, 2012); 197. (Cockerham, 1958); 198. (Solomon-Blackburn and Barker, 2001b); 199. (Solomon-Blackburn and Barker, 2001a); 200. (Marczewski et al., 2001); 201. (Marczewski et al., 2004); 202. (Novy et al., 2007); 203. (Kelley et al., 2009); 204. (Was and Dziewonska, 1984); 205. (Dziewonska and Ostrowska, 1978); 206. (Cockerham, 1955); 207. (Swiezynski et al., 1993); 208. (Marczewski et al., 2006); 209. (Moury et al., 2011); 210. (Brigneti et al., 1997); 211. (Flis et al., 2005); 212. (Mestre et al., 2000); 213. (Mestre et al., 2003); 214. (Barker, 1997); 215. (Jones, 1990); 216. (Barker, 1996); 217. (Valkonen et al., 1995); 218. (Cadman, 1942); 219. (Malcuit et al., 1999); 220. (Cockerham, 1943); 221. (Baerecke, 1967); 222. (Marczewski et al., 1998); 223. (Marczewski et al., 2002); 224. (Fribourg and Nakashima, 1984); 225. (Cruz and Baulcombe, 1993); 226. (Valkonen, 1994); 227. (Tian and Valkonen, 2013); 228. (Szajko et al., 2008); 229. (Hamalainen et al., 1998); 230. (Bendahmane et al., 1995); 231. (Bendahmane et al., 1999); 232. (Bendahmane et al., 2000); 233. (Baures et al., 2008); 234. (Querci et al., 1995); 235. (Munoz et al., 1975); 236. (Bagnall and Young, 1972); 237. (Vidal et al., 2002); 238. (Zvereva and Pooggin, 2012); 239. (Ghazala and Varrelmann, 2007); 240. (Seifers et al., 2012); 241. (Francki et al., 2001); 242. (Stoutjesdijk et al., 2001); 243. (Xu et al., 1999); 244. (Zhang et al., 2000); 245. (Zhang et al., 2001); 246. (Zhang et al., 2004); 247. (Zhang et al., 2009); 248. (Fahim et al., 2012); 249. (Chen et al., 1998a); 250. (Chen et al., 1998b); 251. (Singh, 1993); 252. (Van Koevering et al., 1987); 253. (Zhang et al., 2005); 254. (Modawi et al., 1982); 255. (Hao et al., 2012); 256. (Maiti et al., 2012); 257. (Pal et al., 1991); 258. (Bijaisoradat and Kuhn, 1985); 259. (Ponz et al., 1988); 260. (Sinclair and Walker, 1955); 261. (Kim and Palukaitis, 1997); 262. (Hu et al., 2012); 263. (Karasawa et al., 1999); 264. (Tao et al., 2002); 265. (Palukaitis and Garcia-Arenal, 2003); 266. (Hobbs et al., 1987); 267. (Singh and Singh, 1987); 268. (de Zeeuw and Ballard, 1959); 269. (Bruening et al., 1987); 270. (Taiwo et al., 1981); 271. (Patel et al., 1982) ; 272. (Fan et al., 2011); 273. (Bruening, 2011); 274. (Jones et al., 2007); 275. (Welz et al., 1998); 276. (Shepherd et al., 2010); 277. (Dussle et al., 2002); 278. (Melchinger et al., 1998); 279. (Quint et al., 2002); 280. (Xia et al., 1999); 281. (Xu et al., 2000); 282. (Ding et al., 2012); 283. (Stewart et al., 2013); 284. (Jones, 2012). 285. (de Ronde et al., 2013). 286. (Peiro et al., Accepted for publication). 287. (Yamaji et al., 2012). 288. (Brotman et al., 2013). 289. (Jin et al., 2014). 290. (Chung et al., 2014).
